# Effect of Sm + Er and Heat Treatment on As-Cast Microstructure and Mechanical Properties of 7055 Aluminum Alloy

**DOI:** 10.3390/ma16134846

**Published:** 2023-07-05

**Authors:** Jue Wang, Faguo Li

**Affiliations:** School of Materials Science and Engineering, Xiangtan University, Xiangtan 411105, China; wj200204032022@163.com

**Keywords:** 7055 aluminum alloy, Sm, Er, heat treatment process, fine grain strengthening

## Abstract

The 7055 aluminum alloy is an ultra-high strength aluminum alloy, which is widely used in the aerospace field and new energy automobile manufacturing industry. As it retains high strength, its plastic deformation ability needs to be improved, which limits its application in plastic processing. In this study, the cast grains of the 7055 aluminum alloy were refined by adding Sm + Er, and the proper heat treatment procedure was utilized to further precipitate the rare earth phase in order to increase the alloy’s strength and toughness. The grain size, microstructure and phase were characterized by optical microscopes (OMs), scanning electron microscopy—energy spectrum (SEM-EDS) and a XRD diffractometer (XRD). The macroscopic hardness, yield strength and tensile strength of alloy materials were measured by a hardness meter and universal electronic tensile machine. The results showed that the as-cast sample and the heat treatment sample all contained Al_10_Cu_7_Sm_2_ and Al_8_Cu_4_Er rare earth phases. But, after heat treatment, the volume percentage of the rare earth phase dramatically increased and the dispersion was more unified. When 0.3 wt.%Sm and 0.1 wt.%Er were added, the grain size could be refined to 53 μm. With the increase in the total content of rare earth elements, the refining effect first increased and then decreased. Under 410 °C solid solution for 2 h + 150 °C and aging for 12 h, the macroscopic hardness, yield strength, tensile strength and elongation of 0.3 wt.%Sm + 0.1 wt.%Er + 7055 as-cast samples were 155.8 HV, 620.5 MPa, 658.1 MPa and 11.90%, respectively. After the addition of Sm and Er elements and heat treatment, the grain refinement effect of 7055 aluminum alloy was obvious and the plastic property was greatly improved under the premise of maintaining its high-strength advantage.

## 1. Introduction

The 7055 aluminum alloy is widely used in the aerospace field because of its excellent properties such as high tensile strength and low density [1]. However, over time, the requirements for aircraft performance have become more and more stringent, which has prompted researchers to seek ways to improve the mechanical properties of 7055 aluminum alloy. Through continuous exploration and research, researchers have found that it is possible to improve the microstructure and properties of aluminum alloys [2,3] by various heat treatment processes [4], which change the composition of alloying elements and microalloying elements. Adding Sc, Er, Sm, La, Ce, Y, Zr and other rare earth elements could change the microstructure of aluminum alloy and improve its comprehensive mechanical properties.

The addition of Sc in 7055 aluminum alloy could cause grain boundary effect [5,6], effectively refining the grains, which has a good promoting effect on the hardness, tensile strength, ductility and thermal stability [7,8,9]. But its high cost is not conducive to large-scale industrial applications. In recent years, some scholars have studied the effects of rare earth Ce and La on the microstructure and properties of Al-Zn-Mg-Cu-Zr alloy. They found that Ce and La shortened the distance between the dendrites, which refined the intercrystalline eutectic phase. A small amount of Ce also made the precipitated phase smaller, which improved the mechanical properties of the alloy [10]. Although Ce increased the recrystallized alloy grains and refined the GP region, it had no effect on the properties of the alloy. No precipitated phases such as Al_3_(Zr,Sc) were observed after heat treatment of alloys with La and Ce [11]. When the Zr content reached 0.20 wt.%, the grain refinement effect was the best and the distance between secondary dendrite arms was the smallest. The ultimate tensile strength, yield strength and elongation at room temperature reached the maximum [12]. When Zr content was within a certain range, a fine Al_3_Zr dispersion phase could be formed, which increased the number of crystal nuclei and refined grains [13]. However, after solid solution treatment, the coherent interface between Al_3_Zr particles and the matrix changed to a semi-coherent interface, which led to non-uniform nucleation of solute atoms during quenching and improved the quenching sensitivity [14]. The addition of Y element could greatly improve the grain refinement degree and dislocation density. The formation of β-Al_3_Y intermetallic phase at the grain boundary could not only promote nucleation but also prevent grain growth, so that Al-7.5% (mass fraction) Y alloy could maintain fine grains in the annealed state. However, its effect on grain refinement was not very significant, and the cost performance was not high [15]. In contrast, Sm and Er could form rare earth phases in aluminum alloy and were cheaper than Sc. The rare earth phase could fix the grain boundary during solid solution treatment, and the rare earth atoms could provide more nucleation sites for the precipitation of the η’ phase, which make it smaller during the aging treatment, thus refining the grain [16,17,18]. At the same time, the strongest strengthening effect was present in Er, which effectively prevented grain boundary displacement during its melting process and significantly raised the tensile strength and yield strength of aluminum alloy [19]. The refinement effect of cast aluminum alloy grain was improved after the addition of Sm and it had a considerable effect on the enhancement of plasticity [20]. The compound addition of Sm + Er in 7055 aluminum alloy has potential advantages in improving strength and toughness.

By optimizing the process parameters of heat treatment, homogenizing the alloy composition and adjusting the size and distribution of the second phase and precipitated phase, the comprehensive properties of aluminum alloy could be further improved [21]. Solution aging promoted the precipitation of Al_3_(Er,Zr) particles, which could inhibit the movement of grain boundaries, fix the dislocation and maintain the subgrain structure, so as to effectively improve the comprehensive properties of the material [22,23]. In particular, the precipitation enhancement of Al_3_Er or Al_3_(Er,Zr) particles could compensate for the precipitation enhancement of η’ and play a major role in improving the hardness [24]. Similarly, heat treatment in a certain manner could promote the precipitation of the Sm-containing phase, that is, the formation of Al_2_Sm particles. The Al_2_Sm particles enhanced heterogeneous nucleation, and the grains could be efficiently refined [25].

Solid solution treatment could promote the gradual dissolution of the second phase, thus reducing the resistance of dislocation movement during heat treatment and improving the plasticity of the alloy to a certain extent [26,27]. The main strengthening phase of the alloy treated at 350 °C was the GP region. When the solution treatment temperature was raised to about 400℃, the main strengthening phase evolved into η′ phase and GP region. The η′ phase accounted for 50% of the total nanoprecipitated phase, which was much higher than the proportion of precipitated phase after 350 °C solution aging. Furthermore, the tensile strength and elongation were also greatly improved [28]. With the increase in solution temperature, the size of the precipitated phase would gradually increase in a certain range and the properties of the alloy would be reduced. Under the comprehensive consideration of mechanical properties, the tensile strength was significantly increased and the plastic property was not significantly decreased when the aging temperature was 150 °C [29]. It could maintain the advantages of ultra-high strength of aluminum alloy which prevented the low plastic property and restricted its application. In order to avoid the coarsening of the second phase caused by excessive temperature and make the rare earth phase effectively improve the microstructure and mechanical properties of the material, we carried out a study on the influence of heat treatment processes at 410 °C solution temperature and 150 °C aging temperature on the microstructure and mechanical properties of as-cast 7055 aluminum alloy with Sm + Er composite addition.

## 2. Experimental Method

### 2.1. Sample Composition

The raw materials used in the experiment were 7055 aluminum alloy (Al-8.2Zn-2.2Mg-2.4Cu-0.2Zr), high-purity Sm ingots (99.99 wt.%), high-purity Er ingots (99.99 wt.%), high-purity aluminum foil (99.99 wt.%), etc. The alloy material was weighed by an electronic balance (Shanghai Huachao Industrial Co., LTD., Shanghai, China) with an accuracy of 0.0001 g, and the oxide layer on the surface of the alloy material was polished with 1000 mesh sandpaper before weighing. According to the mass fraction of Sm:Er = 3:1 added to 7055 aluminum alloy, the sum of the added rare earth elements was 0.2–0.8 wt.%. The specific sample composition is shown in Table 1.

### 2.2. Melting Sample

**Figure 1:**
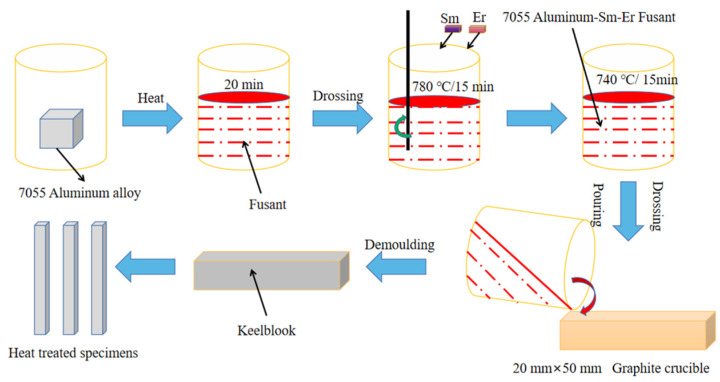
Schematic diagram of smelting, casting and sample preparation process of 7055 aluminum alloy with composite Sm + Er.

The 7055 aluminum alloy with different rare earth compositions was smelted and cast. The temperature of the pit furnace (Xiangtan Samsung Instrument Co., Ltd., Xiangtan, China) was raised to 780 °C for insulation. The 7055 aluminum ingots were loaded into an alumina crucible and put into the pit furnace, and argon gas was used to protect them. When the 7055 aluminum ingots were melted, the rare earth elements were wrapped in aluminum foil during the smelting process, which were pressed into 7055 aluminum alloy melt with a ceramic rod and stirred evenly. After holding for 15 min, the temperature was reduced to 740 °C for refining, so that the gas in the melt was removed to prevent the formation of pores and slag inclusion in the casting. Finally, after holding for 15 min, the crucible was taken out and cast along the mold wall. When the ingot was cooled to room temperature, it was taken out and was wire cut. Details of the smelting, casting and sample making processes are shown in Figure 1.

### 2.3. Heat Treatment

**Figure 2:**
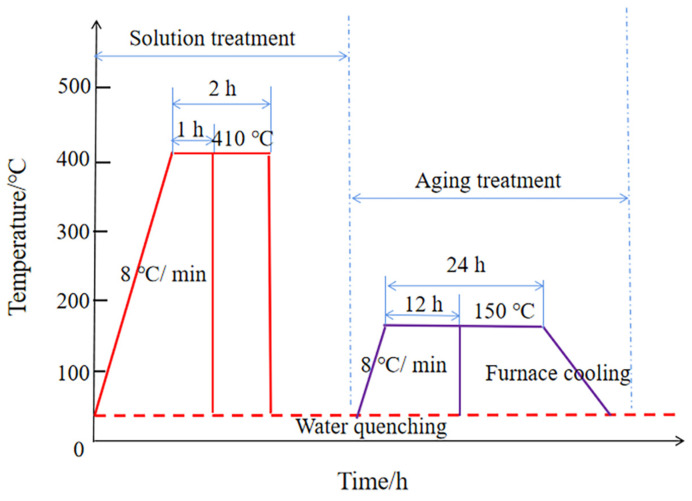
Heat treatment process diagram.

As-cast 7055 aluminum alloy with Sm + Er was treated with single-stage solution and single-stage aging in this experiment. We selected the solution temperature of 410 °C and the aging temperature of 150 °C and we set the solution time to 1 h or 2 h and the aging time to 12 h or 24 h, which formed four different heat treatment processes. The four groups of heat treatment processes were performed as follows: 410 °C × 1 h solid solution treatment + 150 °C × 12 h aging treatment, 410 °C × 1 h solid solution treatment + 150 °C × 24 h aging treatment, 410 °C × 2 h solid solution treatment + 150 °C × 12 h aging treatment and 410 °C × 2 h solid solution treatment + 150 °C × 24 h aging treatment. The heat treatment process diagram is shown in Figure 2.

### 2.4. Characterization Test

**Figure 3:**
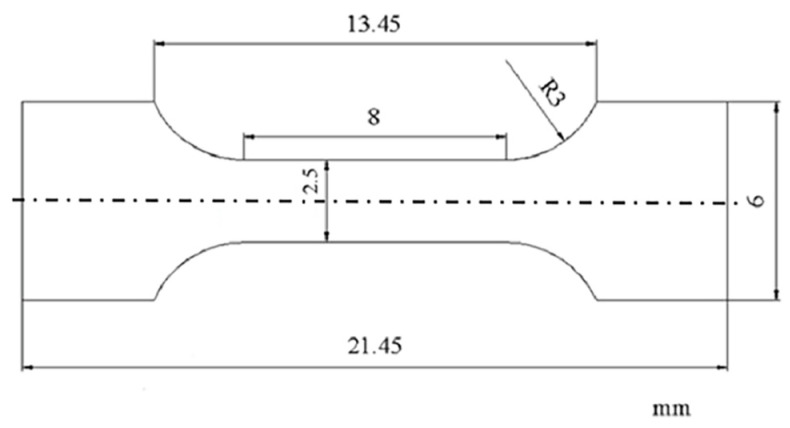
Dimension of tensile drawing.

An OM (ZEISS, Zeiss, Jena, Germany), SEM-EDS (ZEISS, EVO MA10, Zeiss, Jena, Germany) and XRD (Ultima IV, Rigaku Co., Tokyo, Japan) characterized the grain size, micro-organization and phase composition of the as-cast and heat-treated samples. The etching agent for the metallographic sample was Keller reagent (95 mL water, 2.5 mL HNO_3_, 1.5 mL HCl, 1.0 mL HF). The intercept method was used to measure grain size. First, a series of linear segments with the same grain number span were drawn on the microstructure photos, and then the average length of the segments was divided by the grain number. The macroscopic hardness (SHYCHVT-30, Laizhou Huayin Hardness Meter Factory, Lai Zhou China) was measured. First, the plate sample was cut into pieces. Then, it was pre-ground and polished and observed under the Vickers hardness tester microscope. We avoided the strengthening phase and measured the hardness of the substrate. The middle part of the cuboid sample was measured three times, and the macroscopic hardness value was obtained by taking the average value. The penetrator type was diamond pyramid penetrator, which used a force of 0.5 Kg and had a load holding time of 15 s. The tensile samples shown in Figure 3 were prepared by the wire-cut method. The yield strength and tensile strength of the samples were measured by an electronic multifunctional tensile machine (WDW-100C, Jinan Fangyuan Instrument Co., Ltd., Jinan, China). The thickness of the tensile test sample was 1.5 mm and the experimental tensile rate was 0.1 mm/min. The average value was measured three times for each group. The maximum load of the WDW-100C electronic universal tensile testing machine is 100 KN, the test force measurement range is 0.4~100%F.S., and the displacement resolution is 0.002 mm.

## 3. Experimental Results and Analysis

### 3.1. Microscopic Morphology and Physical Phase Analysis of the Alloys

**Figure 4:**
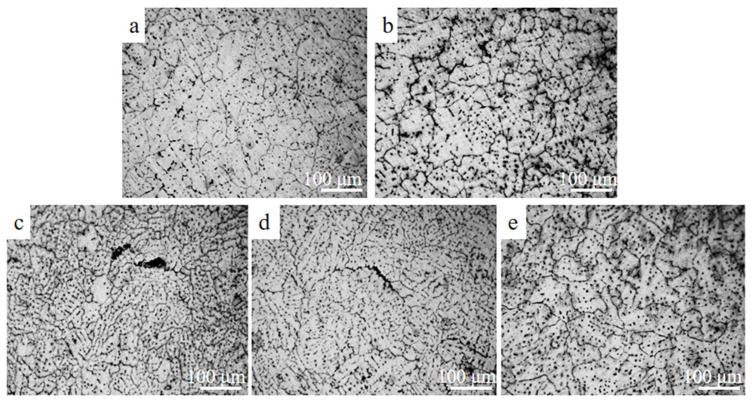
Map of optical microscopy of cast 7055 aluminum alloy with different rare earth contents: (**a**) none; (**b**) 0.2 wt.%; (**c**) 0.4 wt.%; (**d**) 0.6 wt.%; (**e**) 0.8 wt.%.

As can be seen from Figure 4, the grain size of the as-cast 7055 aluminum alloy without adding Sm + Er was 119 μm. The grain size of the as-cast 7055 aluminum alloy was obviously refined by adding Sm + Er. When the total contents of Er and Sm increased from 0.2 to 0.8 wt.%, the grain sizes were 122 μm, 53 μm, 88 μm and 95 μm, respectively. With the increase in the total content of Er and Sm, the grain refining effect first increased and then decreased. The grain refining effect was most obvious when the total content reached 0.4 wt.%. The addition of Sm + Er caused the composition at the nucleation position to be supercooled [30], which increased the nucleation rate and reduced the as-cast grain size. At the same time, the precipitation of rare earth phase containing Sm effectively inhibited the grain boundary movement and refined grains, which greatly improved the plastic properties of aluminum alloy [31]. However, when the added content exceeded a certain amount, a large amount of Er aggregated to form coarse compounds and existed in the grain boundaries due to the low solubility of Er in the Al substrate, which coarsened the grain boundaries and weakened the undercooling effect of rare earth elements on the alloy components [32]. Therefore, adding a proper amount of Sm + Er could refine the cast structure and improve the uniformity of the cast structure.

**Figure 5:**
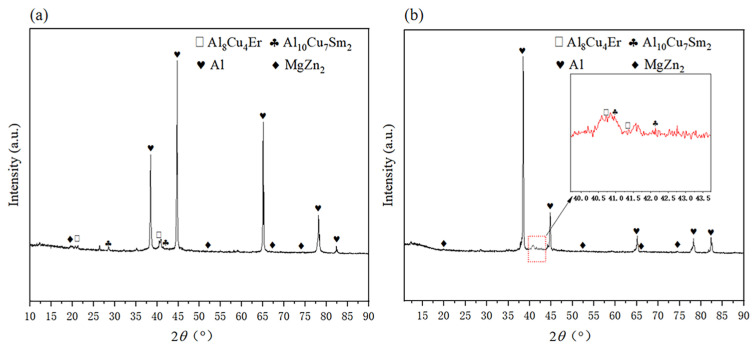
XRD diffraction pattern diagram of sample #2: (**a**) before heat treatment; (**b**) after heat treatment (410 °C × 2 h solution treatment + 150 °C × 12 h aging treatment).

Figure 5 shows the XRD pattern of sample #2 before and after heat treatment. After the addition of rare earth elements Sm + Er, a new Al-RE(-Cu) phase appeared, namely rare earth phases Al_10_Cu_7_Sm_2_ (PDF 36-1267) and Al_8_Cu_4_Er (PDF 33-0006). The diffraction peak of Al_8_Cu_4_Er appeared near 20°, that for Al_10_Cu_7_Sm_2_ near 30° and for both phases around 41°.

**Figure 6:**
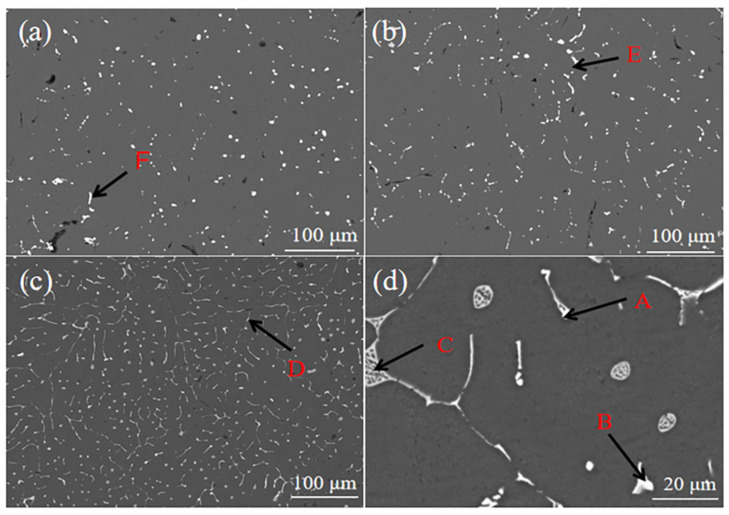
The 7055 SEM micrographs: (**a**) original cast aluminum alloy; (**b**) #2 cast aluminum alloy; (**c**) #2 cast aluminum alloy after 410 °C × 2 h solid solution treatment + 150 °C × 12 h aging treatment; (**d**) the enlarged view of (**c**) (A: Al_10_Cu_7_Sm_2_, B: Al_8_Cu_4_Er, C, D, E, F: α-Al + η-Mg(Zn,Al,Cu)_2_).

Regarding the SEM micro-organization of alloy #2 before and after heat treatment, Table 2 shows the EDS results for each point in Figure 6. According to the EDS results, the atomic ratio of Al, Cu and Sm in the polygon block phase of point A in Figure 6d was close to 10:7:2, which contained a small amount of Zn element. The control XRD result in Figure 5 could be confirmed to be Al_10_Cu_7_Sm_2_. The atomic ratio of Al, Cu and Er in the polygon block phase of point B in Figure 6d was close to 8:4:1, which also contained a small amount of Zn element. The Al_8_Cu_4_Er phase was confirmed by the XRD results in Figure 5.

Figure 6 shows that a large number of coarse-grained alloy phases are distributed at the grain boundaries. After heat treatment (Figure 6c), the precipitation amount of the rare earth phase increased and was uniformly distributed, which had the role of stabilizing the grain boundary. Phases C, D, E and F in Figure 6 were lamellar eutectic structures, which mainly contained Zn, Mg, Al and Cu elements. The XRD results in Figure 5 confirmed the α-Al + η-Mg(Zn,Al,Cu) eutectic microstructure. The crystal structure of η-Mg(Zn,Al,Cu)_2_ was the same as the MgZn_2_ containing few Al and Cu atoms, where a small portion of Al and Cu atoms were soldered [33,34]. When rare earth elements were added, the rare earth phase was preferred over α-Al generation. When α-Al began to nucleate, part of the rare earth phase was pushed to the front end of the solid–liquid interface, thus hindering the further growth of the grain. The lamellar eutectic microstructure in Figure 6c was significantly thinner, which indicated that a large number of second phases at the grain boundary dissolved in the α-Al substrate after heat treatment.

**Figure 7:**
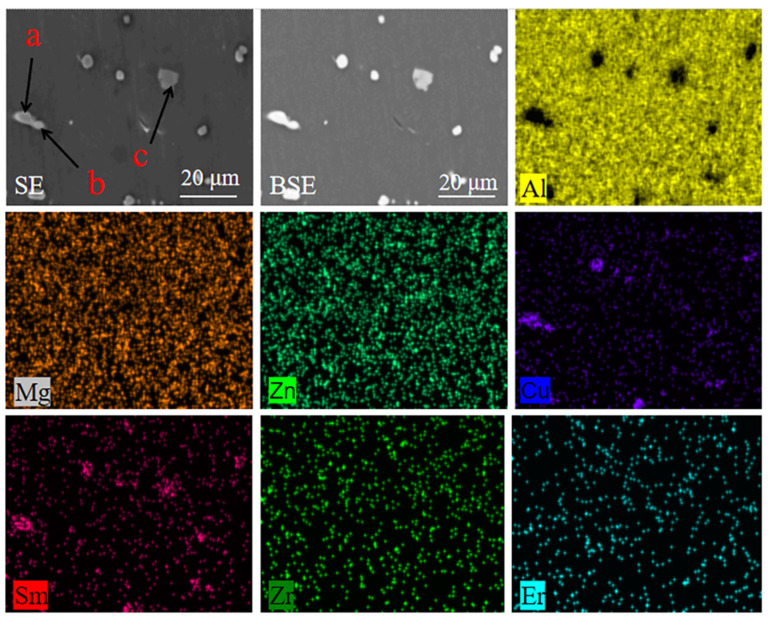
EDS surface scanning results of 2# aluminum alloy after 410 °C × 2 h solid solution treatment + 150 °C × 12 h aging treatment process.

According to the EDS scan results in Figure 7, Cu tended to combine with rare earth elements to form rare earth phases. Sm tended to be enriched in the rare earth alloy phase compared with Er and, furthermore, Mg, Zn and Zr were uniformly dispersed in the substrate.

In summary, the microstructure of as-cast 7055 aluminum alloy could be effectively improved by the addition of Sm + Er, and the mechanical properties of the alloy could be improved by the dispersion of rare earth alloy. The heat treatment process of solution + aging had a significant effect on the microstructure of 7055 aluminum alloy.

### 3.2. Mechanical Property

**Figure 8:**
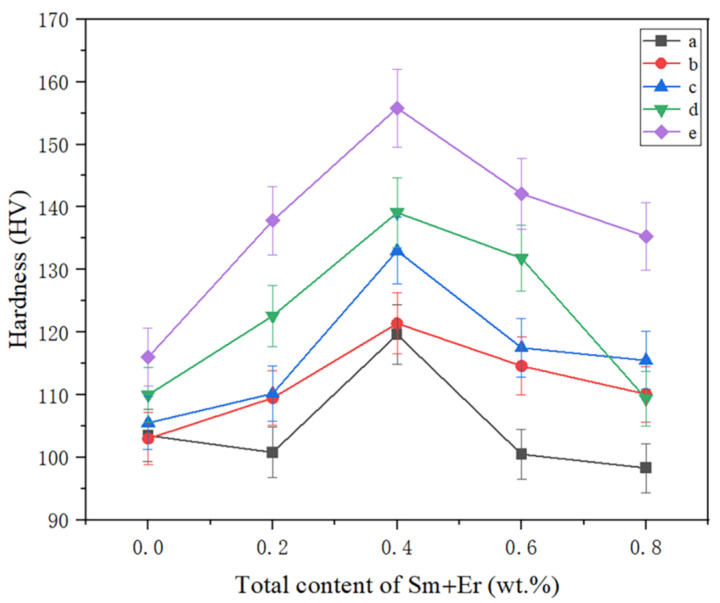
Alloy macroscopic hardness: (a) non-refinement; (b) solid solution 410 °C × 1 h + 150 °C × 24 h; (c) 410 °C × 2 h + 150 °C × 24 h; (d) 410 °C × 1 h + 150 °C × 12 h; (e) 410 °C × 2 h + 150 °C × 12 h.

Figure 8 shows the change in the macroscopic hardness of the four as-cast alloys before and after the four heat treatments. In general, after heat treatment, the hardness of the as-cast alloy increased by 16.7% on average. Among them, the hardness increase in 410 °C × 2 h solid solution treatment + 150 °C × 12 h aging was the highest, which reached 31.4%. The hardness of alloy #2 reached 155.8 HV. Regardless of whether there was heat treatment or not, the hardness of the cast alloy increased with the rare earth content, with a trend of increasing first and then decreasing.

**Figure 9:**
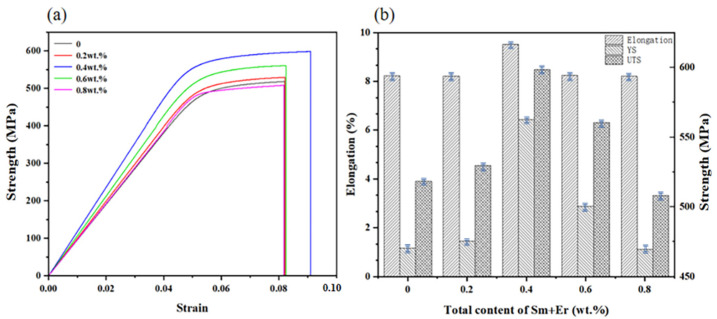

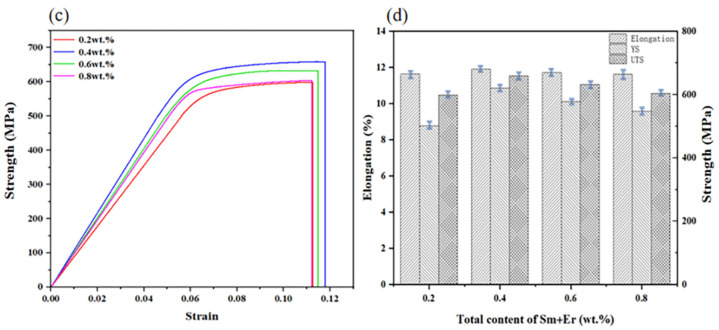
The relationship between the stress and strain curves of different alloys, UTS and elongation: (**a**,**b**) before heat treatment; (**c**,**d**) after heat treatment.

There was a positive relationship between hardness and tensile strength of materials. In this study, various as-cast alloys treated with 410 °C × 2 h solid solution treatment + 150 °C × 12 h aging treatment were selected for comparison of tensile mechanical properties before and after heat treatment. Figure 9 shows the tensile properties of different total contents of Sm + Er before and after heat treatment. It was obvious that the mechanical properties of 7055 aluminum alloy before and after heat treatment were generally improved. The tensile strength, yield strength and elongation were respectively increased by 13.5%, 11.9% and 37.0%. The 7055 aluminum alloy with a total content of 0.4 wt.% Sm + Er reached the maximum tensile strength and yield strength, 658.1 MPa and 620.5 MPa, respectively, and the maximum elongation reached 11.90%. Compared with the original as-cast aluminum alloy, the tensile strength increased by 27.0%, the yield strength increased by 31.9% and the elongation increased by 30.8%. However, with the increase in the content, the alloy strength began to decline and the strengthening effect was significantly weakened. For example, the tensile strength, yield strength and elongation of 7055 aluminum alloy with a total content of 0.8 wt.% Sm + Er before heat treatment decreased by 2.0%, 0.2% and 8.9%, respectively, compared with 7055 aluminum alloy without rare earth elements.

In the 7055 aluminum alloy of the rare earth elements Sm + Er, a certain amount of rare earth phases of Al_10_Cu_7_Sm_2_ and Al_8_Cu_4_Er were precipitated at the grain boundary, inhibiting the movement of the grain boundary and improving the mechanical properties. After heat treatment, the rare earth phase increased and the distribution was more dispersed. The solubility of Er in Al substrate was less than that of Sm, which could effectively inhibit the grain boundary movement and refine the grain [35]. In addition, the Er element provided more nucleation sites for the precipitation of the η phase and made it smaller during the aging process, which effectively inhibited the growth of the lamellar phase and greatly improved the hardness and tensile strength. This can also be seen from the alloy strain uniformity enhancement in Figure 9. As the composite of 7055 aluminum alloy’s average grain size was less than that of 7055 aluminum alloy, it led to the coordinated development of deformation between adjacent grains and timely alleviation of stress concentration. Therefore, it reduced the possibility of crack nucleation, improved the ability of alloy uniform deformation and reduced the influence of defects on the alloy performance [36,37]. When the rare earth content exceeded a certain value, the grain boundary was coarsened, which reduced the effect of rare earth elements on the over-cooling of the alloy, weakening the reinforcement effect.

## 4. Conclusions

By adding different Sm + Er contents to 7055 aluminum alloy, it was found that the rare earth phase formed in the alloy could improve the properties of the alloy. Through a certain heat treatment process, the distribution of the rare earth phase was more uniform and the strength and plasticity of the aluminum alloy were improved. Based on comprehensive analysis, the following conclusions can be drawn:

(1) With the increase in the total amount of Sm + Er, the alloy grain size decreased and then increased. When the total amount reached 0.4 wt.% (0.3 wt.% Sm and 0.1 wt.% Er), the minimum grain size was refined to 53 μm, which had an obvious refinement effect.
(2) The composite addition of Sm + Er produced Al_10_Cu_7_Sm_2_ and Al_8_Cu_4_Er rare earth phases, which could effectively improve the microscopic structure in the solidification process. In the subsequent solid aging treatment, the rare earth phase was more diffusely distributed and the eutectic layer α-Al + η-Mg(Zn,Al,Cu)_2_ also became significantly thinner.
(3) In the solid process (410 °C × 2 h) and aging process (150 °C × 12 h), the mechanical properties of 7055 aluminum alloy with Sm + Er = 0.4 wt.% were the best, which were hardness of 155.8 HV, yield strength of 620.5 MPa, tensile strength of 658.1 MPa, elongation of 11.90%. Compared with the heat treatment, the hardness was increased by 30.3%, the yield strength increased by 10.3%, the tensile strength was increased by 10.0% and the elongation increased by 25.1%.

## Figures and Tables

**Table 1:** Chemical composition of the test samples (wt.%).

Sample Number	Zn	Mg	Cu	Zr	Sm	Er
#1	8.2	2.2	2.4	0.2	0.15	0.05
#2	8.2	2.2	2.4	0.2	0.30	0.10
#3	8.2	2.2	2.4	0.2	0.45	0.15
#4	8.2	2.2	2.4	0.2	0.60	0.20

**Table 2:** EDS results for different points and the corresponding phases in Figure 6 (at.%).

Point	Al	Mg	Cu	Zn	Zr	Sm	Er	Phase/Structure
A	65.08	0	22.40	5.79	0.14	6.59	0	Al_10_Cu_7_Sm_2_
B	55.30	0	30.50	6.68	0.20	0	7.32	Al_8_Cu_4_Er
C	64.03	13.91	4.84	16.21	0.11	0	0	α-Al + η-Mg(Zn,Al,Cu)_2_
D	60.82	18.69	4.94	15.43	0.12	0	0	α-Al + η-Mg(Zn,Al,Cu)_2_
E	56.96	18.34	6.12	17.55	0.13	0	0	α-Al + η-Mg(Zn,Al,Cu)_2_
F	59.78	18.28	6.25	15.60	0.09	0	0	α-Al + η-Mg(Zn,Al,Cu)_2_

## Data Availability

Not applicable.
